# Supplementary material for: Polygenic risk alters the penetrance of monogenic kidney disease
Source: Nat Commun. 2023 Dec 14;14:8318. doi: 10.1038/s41467-023-43878-9 (PMC10721887; doi:10.1038/s41467-023-43878-9)
Supplement: Supplementary file 1 — Supplementary Information [file 41467_2023_43878_MOESM1_ESM.pdf]

Polygenic risk alters the penetrance of monogenic kidney disease

Supplementary Information

Contents

Supplementary Figures

Supplementary Figure 1….. 02
Supplementary Figure 2….. 03
Supplementary Figure 3….. 04
Supplementary Figure 4….. 05
Supplementary Figure 5….. 06
Supplementary Figure 6….. 07
Supplementary Figure 7….. 08

Supplementary Tables

Supplementary Table 1 ….. 09
Supplementary Table 2 ….. 10
Supplementary Table 3 ….. 11
Supplementary Table 4 ….. 12
Supplementary Table 5 ….. 13
Supplementary Table 6 ….. 14
Supplementary Table 7 ….. 15
Supplementary Table 8 ….. 16
Supplementary Table 9 ….. 17
Supplementary Table 10 …..18
Supplementary Table 11 ….. 19
Supplementary Table 12 ….. 20
Supplementary Table 13 ….. 21
Supplementary Table 14 …..22

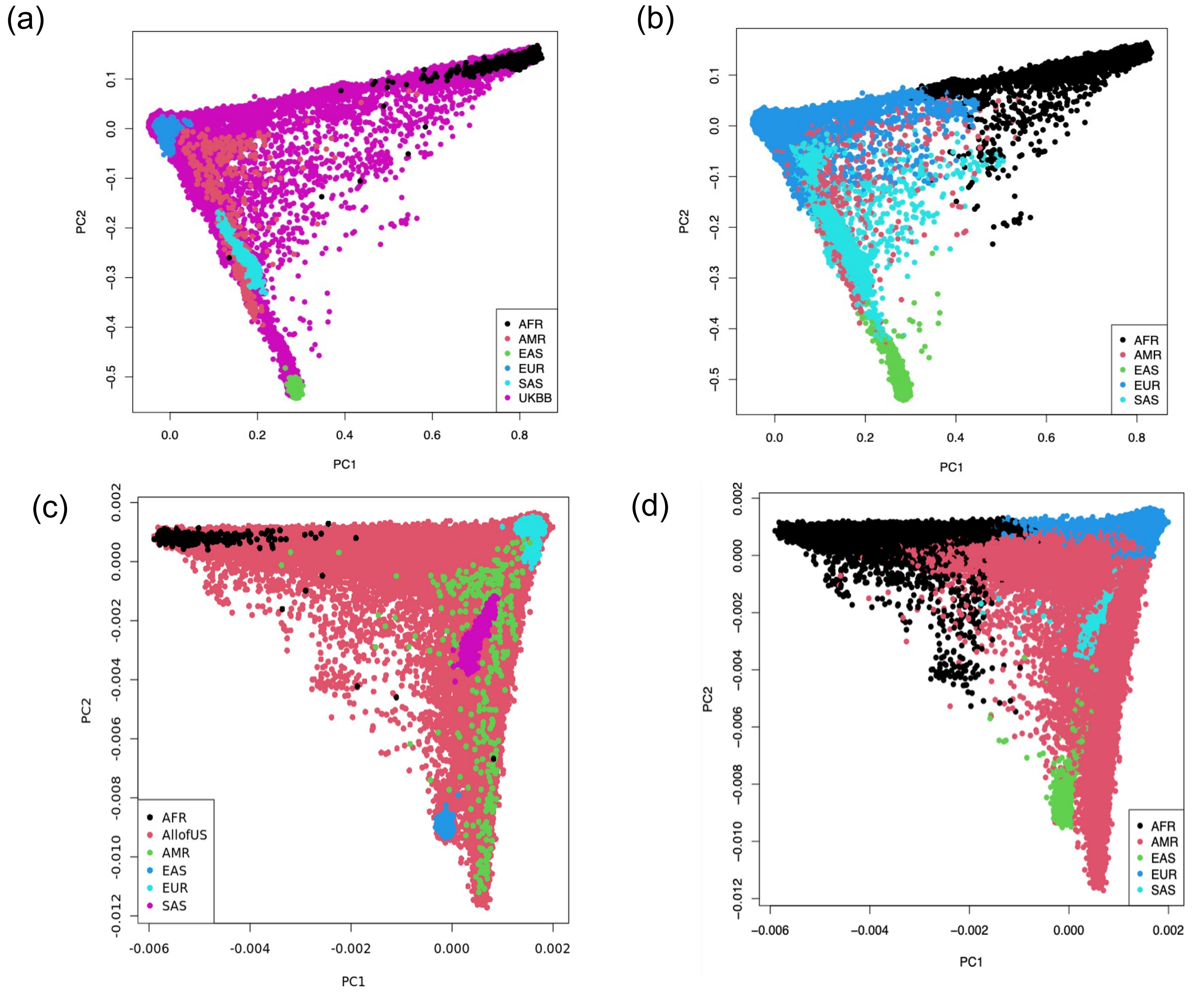

Supplementary Figure 1. PCA projections of the study participants from the UKBB (top) and AoU (bottom) against the 1000 G reference populations: (a) UKBB (N = 460,360) and (c) AoU (N = 165,208) participants plotted against the reference 1000 G populations (N = 2,504), (b) machine learning-assigned ancestry for the UKBB and (d) the AoU datasets. X-axis: PC1; Y-axis: PC2; AFR: African; AMR: Admixed American; EAS: East Asian; EUR: European; and SAS: South Asian.

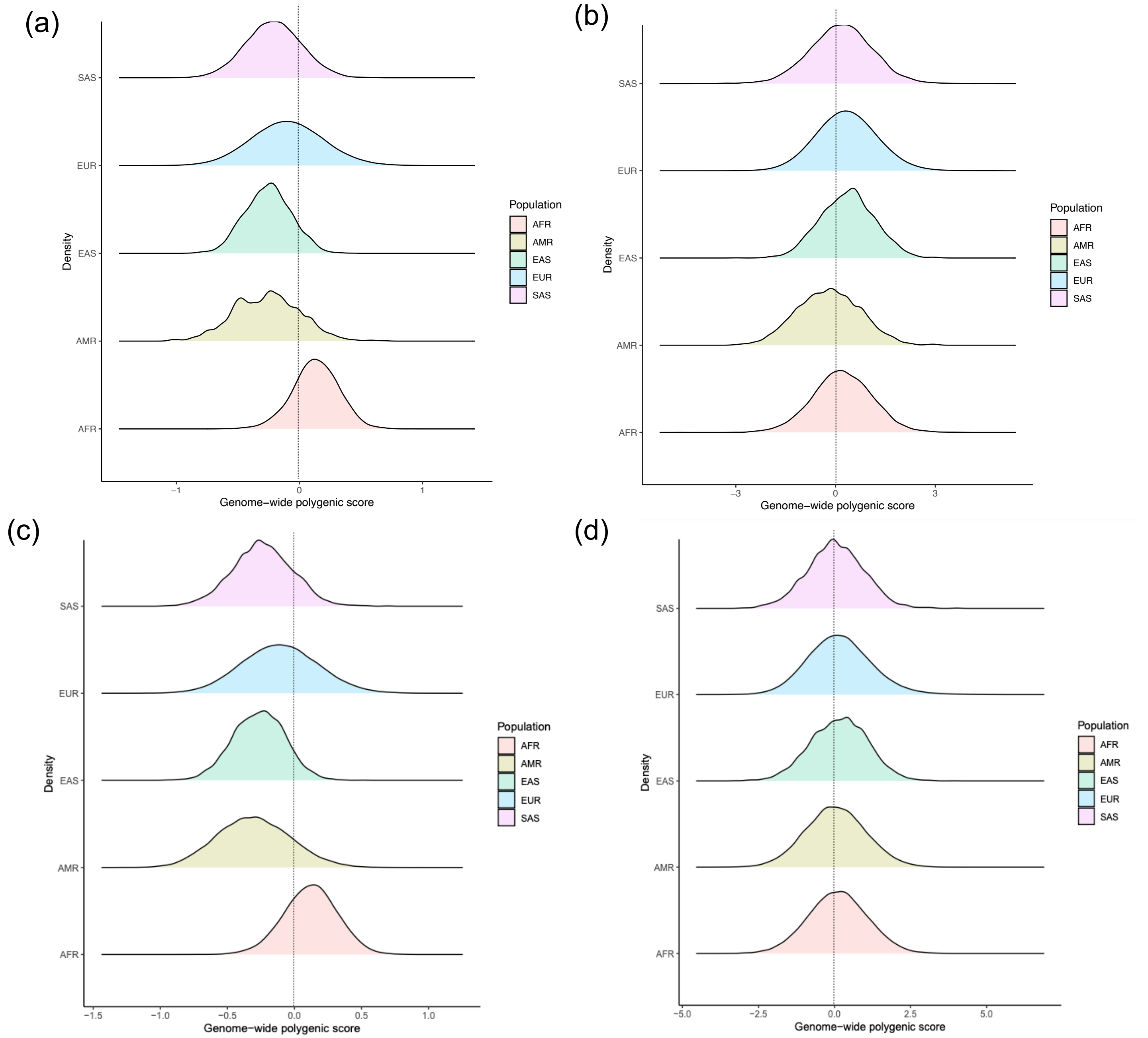

Supplementary Figure 2. Genome-wide Polygenic Score (GPS) distributions by ancestry in the UKBB and AoU datasets: (a) unadjusted and (b) ancestry-adjusted GPS in UKBB (N = 460,360); (c) unadjusted and (d) ancestry-adjusted GPS in AoU (N = 165,208). EUR: European, AFR: African, AMR: Admixed American, EAS: East Asian, and SAS: South Asian.

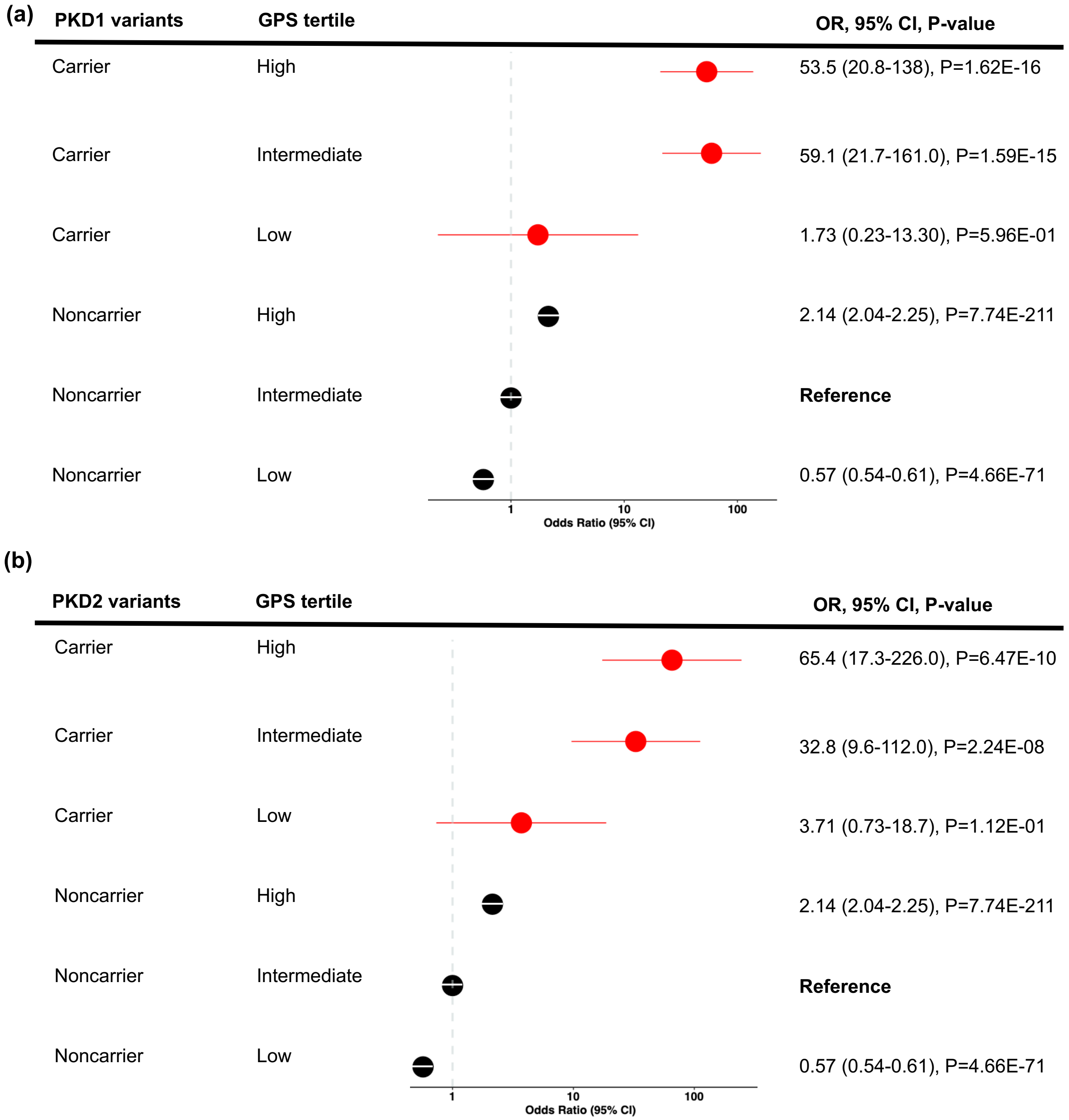

Supplementary Figure 3. Polygenic effects on the risk of CKD in M1 variant carriers for (a) *PKD1* (N_{total}=109) and (b) *PKD2* (N_{total}=63) genes analyzed individually. Each polygenic risk score tertile for carriers was compared to the middle tertile of non-carriers (average population risk). The X-axis shows Odds Ratios (OR), and the dotted vertical line corresponds to the OR=1.0 (no change in risk compared to the reference). The circles correspond to estimated ORs, and the horizontal lines around the circles indicate the 95% confidence intervals (95% CIs). The ORs were estimated using logistic regression model adjusted for age, sex, batch, and ancestry; these estimates were meta-analyzed between AoU and UKBB datasets using fixed effects meta-analysis. Two-sided P-values were derived from fixed effects meta-analysis and were not adjusted for multiple testing.

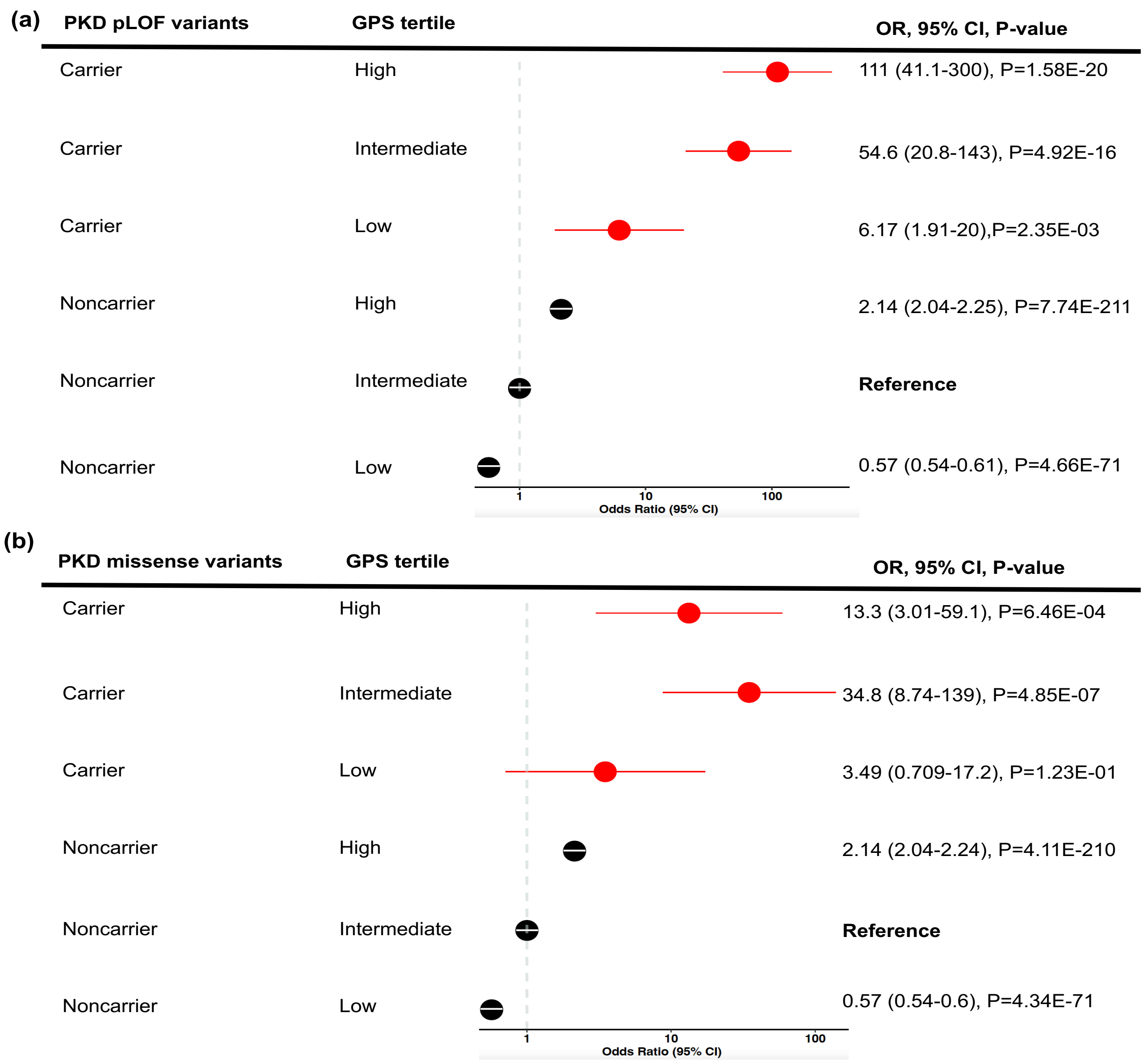

Supplementary Figure 4. Polygenic effects on the risk of CKD in M1 variant carriers for (a) ADPKD pLOF variants (N_{total}=111) and (b) ADPKD missense variants (N_{total}=47) were analyzed individually. Each polygenic risk score tertile for carriers was compared to the middle tertile of non-carriers (average population risk). The X-axis shows Odds Ratios (OR), and the dotted vertical line corresponds to the OR=1.0 (no change in risk compared to the reference). The circles correspond to estimated ORs, and the horizontal lines around the circles indicate the 95% confidence intervals (95% CIs). The ORs were estimated using logistic regression model adjusted for age, sex, batch, and ancestry; these estimates were meta-analyzed between AoU and UKBB datasets using fixed effects meta-analysis. Two-sided P-values were derived from fixed effects meta-analysis and were not adjusted for multiple testing.

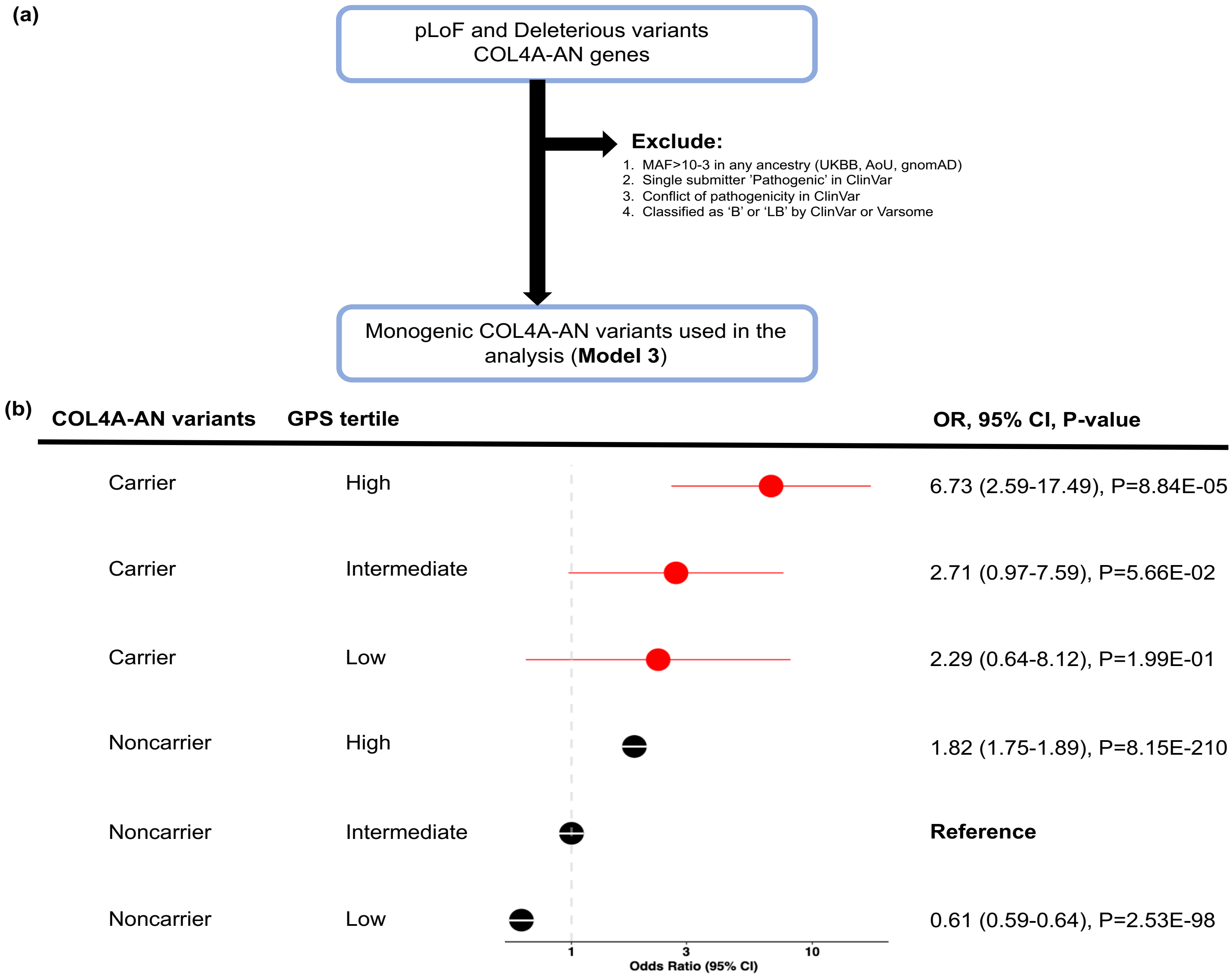

Supplementary Figure 5. Polygenic effects on the risk of CKD among biallelic carriers of COL4A-AN M3 variants (recessive model): (a) M3 qualifying variant filtering strategy; (b) CKD risk for each polygenic risk score tertile compared to the middle tertile in non-carriers (average population risk). The analysis includes N=262,435 UKBB participants (N_{cases}=9,565 and $N_{controls}$=252,870) and N=34,603 AoU participants (N_{cases}=11,830 and $N_{controls}$=22,773). The non-carriers with intermediate polygenic score (middle tertile) served as the reference group for all calculations. The X-axis shows odds ratios; the dotted vertical line corresponds to the OR=1.0 (no change in risk compared to the reference). The circles correspond to estimated ORs, and the horizontal lines around the circles indicate the 95% confidence intervals (95% CIs). The ORs were estimated using logistic regression model adjusted for age, sex, batch, and ancestry; these estimates were meta-analyzed between AoU and UKBB datasets using fixed effects meta-analysis. Two-sided P-values were derived from fixed effects meta-analysis and were not adjusted for multiple testing.

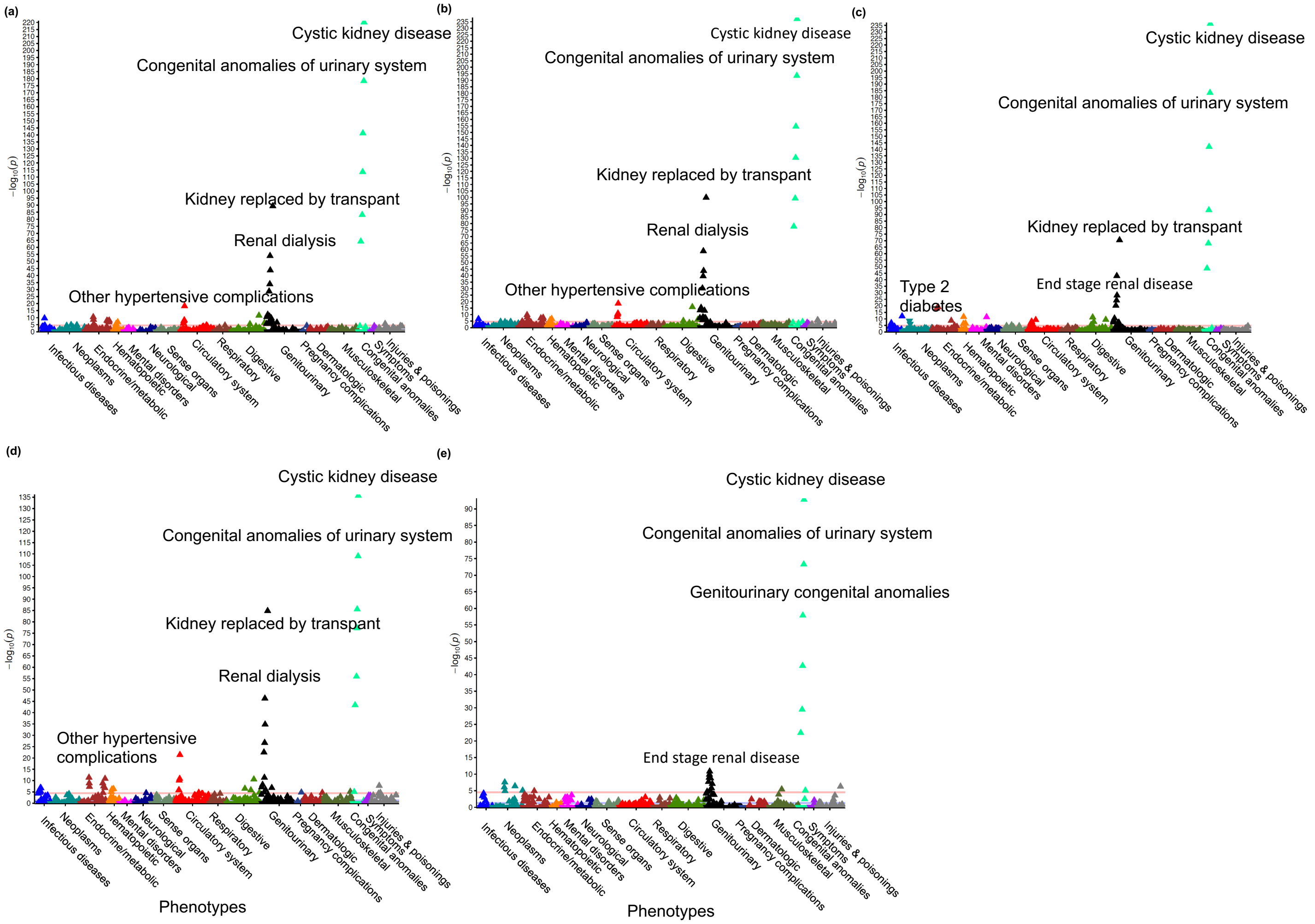

Supplementary Figure 6: ADPKD PheWAS sensitivity analyses in European UKBB participants: (a) M1 model ($N_{carriers}$=136, $N_{non\text{-}carriers}$=416,898), (b) M2 model ($N_{carriers}$=140, $N_{non\text{-}carriers}$=416,898), (c) M3 model ($N_{carriers}$=335, $N_{non\text{-}carriers}$=416,709) (d) *PKD1* gene M1 model ($N_{carriers}$=81, $N_{non\text{-}carriers}$=416,898), and (e) *PKD2* gene M1 model ($N_{carriers}$=55, $N_{non\text{-}carriers}$=416,898). All analyses were conducted using logistic regression under a dominant model with adjustment for age, sex, batch, and genetic ancestry. The red horizontal lines indicate a phenome-wide significance level after accounting for the number of phecodes tested (P=2.8E-05). Y-axis: -log10(P-value). Logistic regression Wald test P-values are two-sided and not adjusted for multiple testing. X-axis: system-based phecode groupings. An upward-pointing triangle indicates increased odds for a given phecode, and a downward-pointing triangle indicates reduced risk.

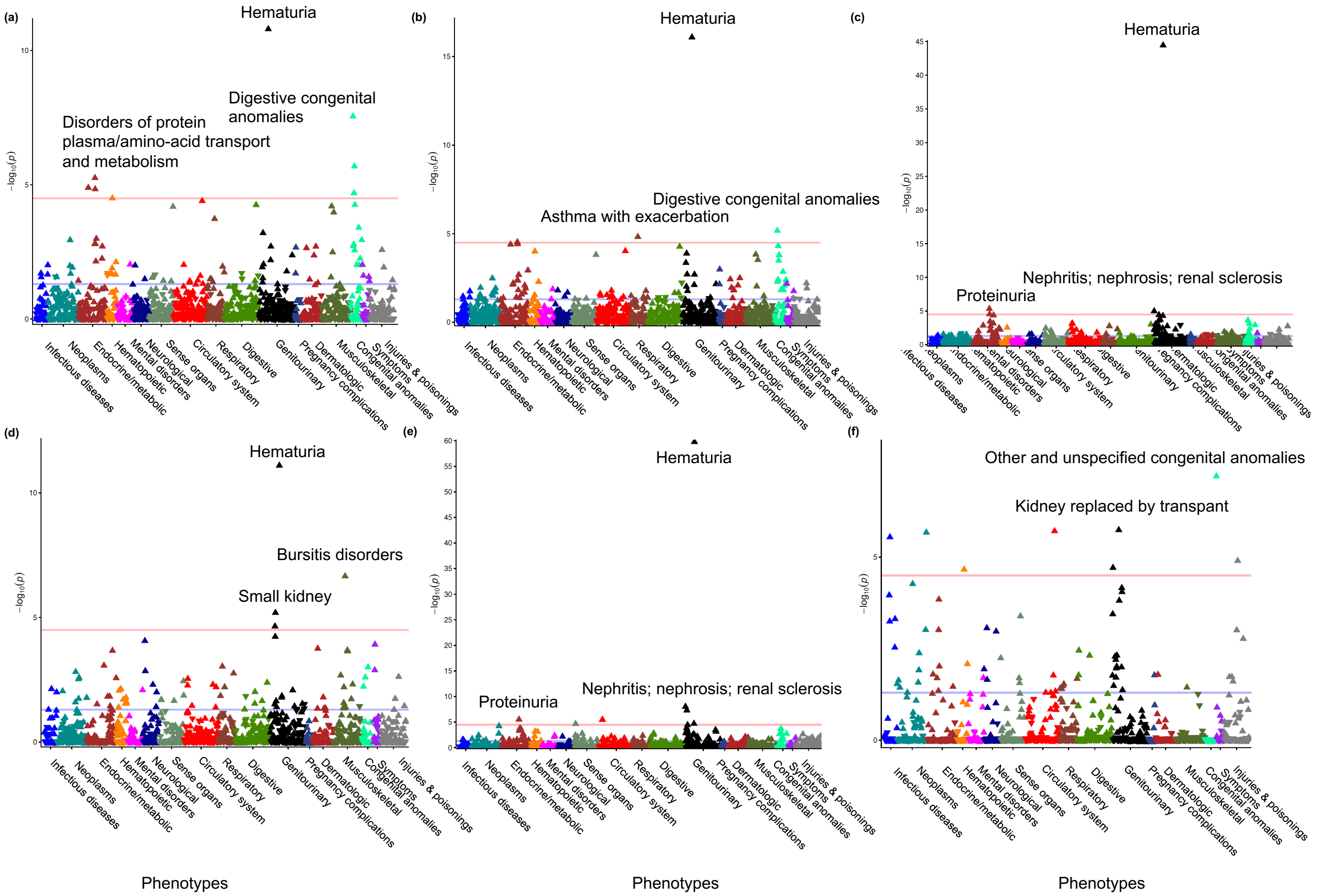

Supplementary Figure 7: COL4A-AN PheWAS Sensitivity Analyses in European UKBB participants: (a) M1 model ($N_{carriers}$=862, $N_{non\text{-}carriers}$=418,068), (b) M2 model ($N_{carriers}$=1,008, $N_{non\text{-}carriers}$=418,020), (c) M3 model ($N_{carriers}$=2,921, $N_{non\text{-}carriers}$=413,938) (d) *COL4A3* gene M1 model ($N_{carriers}$=270, $N_{non\text{-}carriers}$=417,662), (e) *COL4A4* gene M1 model ($N_{carriers}$=505, $N_{non\text{-}carriers}$=417,662) and (f) *COL4A5* gene M1 model ($N_{carriers}$=46, $N_{non\text{-}carriers}$=417,662). All analyses were conducted using logistic regression under a dominant model with adjustment for age, sex, batch, and genetic ancestry. The red horizontal lines indicate a phenome-wide significance level after accounting for the number of phecodes tested (P=2.8E-05). Y-axis: -log10(P-value). Logistic regression Wald test P-values are two-sided and not adjusted for multiple testing. X-axis: system-based phecode groupings. An upward-pointing triangle indicates increased odds for a given phecode, and a downward-pointing triangle indicates reduced risk.

Supplementary Table 1: M1, M2, and M3 variant carriers and their characteristics in the UKBB and AoU datasets. The counts include only individuals with a valid phenotype (case/control) label that was included in the analyses. Note that the non-carrier group is common to all three variant models and excludes any M1, M2, and M3 variant carriers.

Cohorts	N Total	N Cases (%)	N Controls (%)	Female	Diabetes	Mean Age in Years (Range)
UK Biobank	277,165	10,123 (3.6%)	267,042 (96.4%)	54%	6%	56.5 (40-69)
ADPKD M1 Carriers	115	36 (31.3%)	79 (68.7%)	60%	11%	54.6
ADPKD M2 Carriers	125	39 (31.2%)	86 (68.8%)	62%	11%	53.7
ADPKD M3 Carriers	256	45 (17.5%)	211 (82.5%)	56%	7%	54.4
ADPKD Non-carriers	264,158	9,565 (3.6%)	252,870 (96.4%)	55%	6%	54.6
COL4-AN M1 Carriers	1,214	62 (5.1%)	1,152 (94.9%)	55%	7%	54.9
COL4-AN M2 Carriers	1,350	65 (4.8%)	1,285 (95.2%)	55%	7%	54.5
COL4-AN M3 Carriers	1,830	100 (5.4%)	1,730 (94.6%)	57%	7%	54.5
COL4-AN Non-carriers	264,239	9,646 (3.6%)	254,593 (96.4%)	54%	6%	56.5
All of Us	34,603	11,830 (3.41%)	22,773 (96.59%)	60%	11%	54.9 (18-89)
ADPKD M1 Carriers	7	5 (71.4%)	2 (28.6%)	71%	14%	60.5
ADPKD M2 Carriers	14	5 (35.7%)	9 (64.3%)	64%	14%	59.6
ADPKD M3 Carriers	11	7 (63.3%)	4 (36.7%)	73%	27%	64.7
ADPKD Non-carriers	34,588	11,820 (3.4%)	22,768 (96.6%)	60%	11%	54.1
COL4-AN M1 Carriers	78	37 (47.4)	41 (52.6%)	58%	23%	56.0
COL4-AN M2 Carriers	106	47 (44.3%)	59 (55.7%)	65%	21%	54.5
COL4-AN M3 Carriers	226	72 (31.8%)	154 (68.2%)	65%	20%	54.4
COL4-AN Non-carriers	34,539	11,800 (3.4%)	22,739 (96.6%)	60%	11%	54.9

Supplementary Table 2. Imputation of the AoU dataset using phase 3 1000 Genomes project reference panel (all

Chromosomes	Array SNPs	Imputed SNPs (MAF>=0.00 and R2>=0)	Imputed SNPs (MAF=0.001 and R2=0.30)	Imputed SNPs (MAF=0.001 and R2=0.80)	Imputed SNPs (MAF=0.01 and R2=0.80)
1	89688	3738240	2040921	1509280	909349
2	99772	4057613	2231592	1704713	1003370
3	84384	3355939	1879863	1455774	863987
4	76774	3338265	1899659	1474223	876432
5	69273	3032422	1704339	1320209	766074
6	82492	2954410	1694019	1328952	799683
7	63934	2753497	1539955	1158989	694545
8	60000	2651561	1470694	1135036	669886
9	49556	2063096	1121854	842974	505766
10	56241	2334090	1296803	986085	595717
11	56476	2333242	1286023	978430	581944
12	53025	2242720	1256253	945606	572855
13	40542	1661700	944885	726461	434997
14	36097	1535592	841576	636776	383314
15	35262	1404164	757414	555375	335228
16	38698	1549316	822821	591403	358495
17	33636	1345835	721632	495211	307640
18	33408	1319629	739218	551084	334086
19	24882	1084535	585112	379198	247001
20	27606	1047613	577463	420925	258119
21	16391	653791	353514	253922	159090
22	16388	652195	343797	234264	148765
Total	1,144,525	43,371,225	26,109,407	19,684,890	11,806,343

populations): numbers of variants per chromosome before and after imputation by minor allelic frequency (MAF) and imputation quality (R2).

Supplementary Table 3: Genetic ancestry of the AoU dataset based on supervised machine learning with labeled phase 3 1000 Genomes project reference panel for training.

Ancestry	N (%)	Age (Mean in years)	Sex (% Female)
EUR (European)	94,376 (57)	58.82	60.03
AFR (African)	36,380 (22)	51.90	57.68
AMR (Admixed American)	28,807 (17)	47.73	67.89
EAS (East Asian)	3,940 (2)	47.04	63.76
SAS (South Asian)	1,705 (1)	45.76	50.35
Total	165,208	50.25	59.94

Supplementary Table 4: Overall frequencies of *APOL1* G1 and G2 risk alleles and risk genotypes by ancestry and cohort.

Dataset	Ancestry	APOL1- 1072A>G (rs73885319)	APOL1- 1200T>G (rs60910145)	APOL1-1212- del6 (rs71785313)	APOL1 risk genotype (G1G1, G1G2, or G2G2)
UKBB					
	African (AFR)	0.28	0.28	0.15	0.12
	Europeans (EUR)	3.75E-05	1.96E-04	2.91E-05	<0.01
AoU					
	African (AFR)	0.233	0.232	0.139	0.12
	Admixed American (AMR)	0.022	0.022	0.018	0.003
	Europeans (EUR)	7.58E-04	7.58E-04	5.19E-04	<0.01

Supplementary Table 5: Genome-wide Polygenic Score (GPS) Re-optimization. The table summarizes associations of candidate polygenic scores with CKD in the UKBB re-optimization dataset (70% of UKBB Europeans after excluding all ADPKD/COL4A-AN qualifying variant carriers). The performance was assessed using logistic regression with GPS as a predictor and case/control status as an outcome. Two-sided P-values were derived for the Wald test of the GPS effect and were not adjusted for multiple testing. Odds ratio (OR) per standard deviation (SD) of each risk score and area under the receiver-operator curve (AUC) were calculated in the UKBB optimization dataset of 175,835 Europeans excluding QV carriers and after adjustment for age, sex, diabetes, first four principal components of ancestry and genotyping batch; AUC crude was calculated for the risk score component alone without any covariates; variance explained is estimated as a Nagelkerke pseudo-R2 and refers to the variance in case-control status explained by the risk score alone excluding covariate contributions; r2: linkage disequilibrium pruning threshold; rho: tuning parameter to model the proportion of variants assumed to be causal; the best performing score is highlighted in bold red.

Method	Parameter	N variants	OR* per SD of GPS	P-value	AUC (Adjusted*)	AUC (Crude)	Variance Explained
P+T	P=1.0E-01	89,880	1.85	P<1.00E-300	0.8392	0.6508	0.0438
P+T	P=1.0E-02	21,764	1.85	P<1.00E-300	0.8399	0.6518	0.0442
P+T	P=1.0E-03	7,486	1.79	P<1.00E-300	0.8380	0.6446	0.0405
P+T	P=1.0E-04	3,598	1.78	P<1.00E-300	0.8374	0.6422	0.0394
P+T	P=1.0E-05	2,111	1.76	P<1.00E-300	0.8366	0.6396	0.0378
P+T	P=1.0E-06	1,407	1.75	P<1.00E-300	0.8365	0.6390	0.0372
P+T	P=1.0E-07	1,028	1.76	P<1.00E-300	0.8362	0.6388	0.0370
P+T	P=1.0E-08	753	1.74	P<1.00E-300	0.8353	0.6362	0.0355
P+T	P=3.0E-02	41,426	1.86	P<1.00E-300	0.8400	0.6527	0.0450
P+T	P=3.0E-03	11,918	1.83	P<1.00E-300	0.8391	0.6492	0.0428
P+T	P=3.0E-04	4,971	1.79	P<1.00E-300	0.8378	0.6436	0.0401
P+T	P=3.0E-05	2,675	1.76	P<1.00E-300	0.8369	0.6404	0.0385
LDPred	rho=1.0E+00	5,440,627	1.84	P<1.00E-300	0.8387	0.6512	0.0433
LDPred	rho =1.0E-01	5,440,627	1.20	P=2.60E-44	0.8168	0.5472	0.0039
LDPred	rho =1.0E-02	5,440,627	1.16	P=1.03E-28	0.8161	0.5385	0.0025
LDPred	rho =1.0E-03	5,440,627	1.14	P=4.78e-24	0.8157	0.5334	0.0020
LDPred	rho =3.0E-01	5,440,627	1.72	P<1.00E-300	0.8339	0.6348	0.0342
LDPred	rho =3.0E-02	5,440,627	1.12	P=1.05E-18	0.8155	0.5287	0.0016
LDPred	rho =3.0E-03	5,440,627	1.16	P=5.98E-31	0.8161	0.5385	0.0027

* Adjusted for age, sex, diabetes, the first four principal ancestry components and genotyping batch.

Supplementary Table 6: The effect of ADPKD qualifying variant (QV) carrier status on the risk of CKD in the UK Biobank and the All of Us datasets. The Odds Ratios (ORs) were derived from logistic regression adjusted for age, sex, diabetes, batch, and genetic ancestry and were combined across both datasets using fixed effects model. Two-sided P-values correspond to the association tests of carrier status as a predictor of CKD (logistic regression Wald test or fixed effects meta-analysis for combined datsets) and are not corrected for multiple testing; M1 includes only pLOF, and 'P' variants (N_{total}=122 carriers); M2 includes pLOF, 'P', and 'LP' variants (N_{total}=139 carriers); M3 includes pLOF and all deleterious missense variants as defined by 5 prediction algorithms, Revel >0.7, and not previously classified as 'B' or 'LB' by ClinVar (N_{total}=267 carriers). All comparisons are made in reference to the common group of non-carriers (N_{total}=297,539). CI: Confidence Intervals.

	ADPKD M1 carriers<br>OR (95%CI), P	ADPKD M2 carriers<br>OR (95%CI), P	ADPKD M3 carriers<br>OR (95%CI), P
UKBB	18.2 (11.5-28.6), P=5.1E-36	17.3 (11.1-27.0), P=4.8E-36	7.1 (4.95-10.2), P=1.8E-26
AoU	8.36 (1.84-37.4), P=5.9E-03	6.11 (1.47-25.4), P=1.3E-02	3.13 (0.70-14.1), P=1.4E-01
Meta-analysis	17.1 (11.1-26.4), P=1.8E-37	15.8 (10.3-24.2), P=5.2E-37	6.77 (4.76-9.60), P=1.0E-26

Supplementary Table 7: Associations of GPS and ADPKD M1 carrier status with CKD stage 3 or above in the UKBB and All-of-Us datasets. All analyses were performed using logistic regression and were adjusted for age, sex, diabetes, batch, and genetic ancestry. The estimates from individual cohorts were combined using fixed effects meta-analysis. Two-sided P-values correspond to the logistic regression Wald test (or fixed effects meta-analysis for combined datasets) and are not corrected for multiple testing. NS: not significant.

Dataset	Cases/controls	CKD GPS OR per SD (95% CI), P	M1 ADPKD carrier OR (95% CI), P	GPS by M1 carrier interaction OR (95% CI), P
UKBB	10,081/266,724	1.80 (1.76-1.84), P< E-300	18.2 (11.5-28.6), P=5.08E-36	1.43 (0.76-2.72), P=2.68E-01 (NS)
AoU	11,820/22,763	1.40 (1.36-1.44), P=8.52E-211	8.36 (1.8-37.3), 5.8E-03	1.04 (0.15-7.03), P=6.68E-01 (NS)
Meta	21,901/289,487	1.72 (1.69-1.76), P<E-300	17.0 (11.0-26.4), P=1.82E-37	1.39 (0.76-2.56), P=2.87E-01 (NS)

Supplementary Table 8: Genome-wide polygenic score (GPS) performance metrics among ADPKD M1, M2, and M3 carriers and non-carriers. The odds ratios (ORs) were first derived using logistic regression models adjusted for age, sex, diabetes, batch, and genetic ancestry for each individual cohort (UKBB and All-of-Us) followed by a fixed-effect meta-analysis of both cohorts. All effect estimates were calculated in reference to the middle tertile of non-carriers (average risk). Two-sided P-values correspond to the fixed effects meta-analysis and are not corrected for multiple testing.

Model	Cases/Controls	OR per SD (95% CI), P-value	GPS Tertile	OR (95% CI), P-value
Noncarrier				
	21,901/275,638	1.72 (1.69-1.76), P<E-300	Tertile 1	0.62 (0.59-0.65), P=3.9E-96
			Tertile 2	Reference
			Tertile 3	1.82 (1.75-1.89), P=3.4E-208
M1				
	41/81	2.28 (1.55-3.37), P=2.7E-05	Tertile 1	3.03 (1.03-8.95), P=4.4E-02
			Tertile 2	35.8 (16.8-76.4), P=2.0E-20
			Tertile 3	54.4 (26.2-113.1), P=9.6E-27
M2				
	44/95	2.21 (1.37-3.58), P=3.3E-05	Tertile 1	4.99 (1.94-12.8), P=8.6E-04
			Tertile 2	24.1 (11.5-50.5), P=3.0E-17
			Tertile 3	49.4 (24.0-101.6), P=3.1E-26
M3				
	52/215	5.25 (2.31-11.9), P=7.4E-05	Tertile 1	1.89 (0.77-4.63), P=1.6E-01
			Tertile 2	8.52 (4.73-15.3), P=8.8E-13
			Tertile 3	21.8 (12.4-38.1), P=4.7E-27

Supplementary Table 9: Sensitivity analyses comparing race-free (2021) vs. race-adjusted (2009) CKD-EPI eGFR equations in the UKBB. GPS effects on the risk of CKD stage 3 or above for (a) ADPKD and (b) COL4A-AN M1 variant carriers. All effect estimates were calculated using logistic regression in reference to the middle tertile of non-carriers (average risk) and were adjusted for age, sex, diabetes, batch, and genetic ancestry. Case-control counts are provided for each analysis as defined by the CKD-EPI 2021 and 2009 equations. Two-sided P-values (logistic regression Wald test) were not corrected for multiple testing.

Dataset	Phenotype	Cases/controls	OR (95% CI), P-value	GPS Tertile	OR (95% CI), P-value
(a) ADPKD M1 model					
	CKD-EPI 2021 (New) Equation	33/86	2.43 (1.34-4.39), P=3.3E-03	Tertile 1	4.81 (1.43-16.1), P=1.07E-02
				Tertile 2	49.9 (23.4-106), P=3.94E-24
				Tertile 3	64.1 (31.1-132), P=1.53E-29
	CKD-EPI 2009 (Race-adjusted) Equation	36/79	2.45 (1.37-4.38), P=2.6E-03	Tertile 1	2.71 (0.78-9.37), P=1.2E-01
				Tertile 2	40.4 (18.3-89.3), P=5.9E-20
				Tertile 3	59.7 (28.3-126), P=9.2E-27
(b) COL4A-AN M1 model					
	CKD-EPI 2021 (New) Equation	43/1,284	2.53 (1.52-4.22), P=3.4E-04	Tertile 1	1.38 (0.67-2.84), P=3.8E-01
				Tertile 2	1.70 (0.86-3.36), P=1.2E-01
				Tertile 3	2.77 (1.59-4.85), P=3.4E-01
	CKD-EPI 2009 (Race-adjusted) Equation	62/1,152	1.93 (1.26-2.95), P=2.3E-03	Tertile 1	1.10 (0.57-2.12), P=7.7E-01
				Tertile 2	1.45 (0.82-2.55), P=1.9E-01
				Tertile 3	2.77 (1.73-4.46), P=2.6E-05

Supplementary Table 10: Sensitivity analysis of the UKBB participants of European ancestry by eGFR equation. GPS effects on the risk of CKD in the (a) ADPKD and (b) COL4A-AN M1 variant carriers. All effect estimates were calculated using logistic regression in reference to the middle tertile of non-carriers (average risk) and were adjusted for age, sex, diabetes, batch, and genetic ancestry. Case-control counts are provided for each analysis as defined by the CKD-EPI 2021 and 2009 equations. Two-sided P-values (logistic regression Wald test) were not corrected for multiple testing.

Dataset	Phenotype	Cases/controls	OR (95% CI), P-value	GPS Tertile	OR (95% CI), P-value
(A) ADPKD					
	CKD-EPI 2021 (New) Equation	28/65	2.48 (1.30-4.72), P=5.6E-03	Tertile 1	3.86 (0.89-16.7), P=7.1E-02
				Tertile 2	58.0 (25.6-131), P=2.3E-22
				Tertile 3	96.5 (42.7-218), P=4.6E-28
	CKD-EPI 2009 (Race-adjusted) Equation	31/63	2.52 (1.35-4.73), P=3.9E-03	Tertile 1	1.82 (0.41-8.02), P=4.3E-01
				Tertile 2	44.3 (18.8-104), P=4.01E-18
				Tertile 3	78.3 (32.6-188), P=1.4E-22
(B) COL4A-AN					
	CKD-EPI 2021 (New) Equation	41/1240	2.51 (1.51-4.17), P=4.0E-04	Tertile 1	1.25 (0.58-2.69), P=5.7E-01
				Tertile 2	1.76 (0.89-3.47), P=1.0E-01
				Tertile 3	2.64 (1.48-4.71), P=9.8E-04
	CKD-EPI 2009 (Race-adjusted) Equation	60/1109	1.94 (1.26-2.98), P=2.7E-03	Tertile 1	1.03 (0.51-2.04), P=9.4E-01
				Tertile 2	1.47 (0.83-2.59), P=1.8E-01
				Tertile 3	2.75 (1.69-4.46), P=4.5E-05

Supplementary Table 11: The effect of COL4A-AN qualifying variant (QV) carrier status on the risk of CKD in the UKBB and the AoU datasets. All analyses were performed using logistic regression and were adjusted for age, sex, diabetes, batch, and genetic ancestry. The estimates from individual cohorts were combined using fixed effects meta-analysis. Two-sided P-values correspond to the logistic regression Wald test (or fixed effects meta-analysis for combined datasets) and are not corrected for multiple testing; M1 includes only pLOF, and 'P' variants (N_{total}=1,292 carriers); M2 includes pLOF, 'P', and 'LP' variants (N_{total}=1,458 carriers); M3 includes pLOF and all deleterious missense variants as defined by 5 prediction algorithms, Revel >0.7, and not previously classified as 'B' or 'LB' by ClinVar (N_{total}=2,056 carriers); M3 recessive model (N_{total}=127) includes biallelic carriers of M3 variants for COL4A3 or COL4A4, or M3 hemizygous males. All comparisons are made in reference to the common group of non-carriers (N_{total}=298,778).

Datasets	COL4A-AN M1 carriers OR (95%CI), P	COL4A-AN M2 carriers OR (95%CI), P	COL4A-AN M3 carriers OR (95%CI), P	COL4A-AN M3 recessive OR (95%CI), P
UKBB	1.41 (1.15-1.74), P=1.1E-03	1.34 (1.03-1.73), P=3.0E-02	1.55 (1.26-1.92), P=5.0E-05	3.10 (1.66-5.78), P=4.2E-04
AoU	1.21 (0.82-1.79), P=3.3E-01	1.05 (0.67-1.61), P=8.2E-01	1.29 (0.90-1.85), P=1.6E-01	6.69 (1.23-36.4), P=2.8E-02
Meta	1.37 (1.13-1.64), P=8.5E-04	1.25 (1.00-1.56), P=4.9E-02	1.48 (1.23- 1.77), P=2.6E-05	3.38 (1.88-6.08), P=4.7E-05

Supplementary Table 12: COL4A-AN status and the GPS for association with CKD stage 3 or above. All analyses were performed using logistic regression and were adjusted for age, sex, diabetes, batch, and genetic ancestry. The estimates from individual cohorts were combined using fixed effects meta-analysis. Two-sided P-values correspond to the logistic regression Wald test (or fixed effects meta-analysis for combined datasets) and are not corrected for multiple testing. NS: not significant.

Datasets	Cases/controls	CKD GPS OR per SD (95% CI), P	PKD carrier OR (95% CI), P	GPS and COL4A-AN interaction OR (95% CI), P
UKBB				
	16/156	1.80 (1.76-1.84), P< E-300	1.41 (1.15-1.74), P=1.10E-03	0.98 (0.57-1.70), P=9.40E-01 (NS)
AoU				
	5/06	1.40 (1.35-1.44), P=9.18E-85	1.21 (0.82-1.79), P=3.32E-01	0.77 (0.23-2.62), P=6.81E-01 (NS)
Meta	21/162	1.78 (1.22-2.58), P=2.38E-03	1.37 (1.13-1.64), P=8.52E-04	0.94 (0.57-1.55), P=8.12E-01 (NS)

Supplementary Table 13. Genome-wide polygenic score (GPS) performance metrics among COL4A-AN M1, M2, and M3 variant carriers and non-carriers. The odds ratios (ORs) were first derived using logistic regression models adjusted for age, sex, diabetes, batch, and genetic ancestry for each individual cohort (UKBB and All-of-Us) followed by a fixed-effect meta-analysis of both cohorts. All effect estimates were calculated in reference to the middle tertile of non-carriers (average risk). Two-sided P-values correspond to the fixed effects meta-analysis and are not corrected for multiple testing.

Model	Cases/Controls	OR per SD (95% CI), P-value	GPS Tertile	OR (95% CI), P-value
Noncarrier				
	21,446/277,332	1.70 (1.68-1.73), P<E-300	Tertile 1	0.61 (0.59-0.64), P=2.5E-98
			Tertile 2	Reference
			Tertile 3	1.82 (1.75-1.89), P=8.1E-210
M1				
	99/1,193	1.78 (1.22-2.58), P=2.4E-03	Tertile 1	1.08 (0.63-1.86), P=7.7E-01
			Tertile 2	1.66 (1.03-2.68), P=3.7E-02
			Tertile 3	2.53 (1.66-3.85), P=1.4E-05
M2				
	112/1,344	2.47 (1.56-3.94), P=1.3E-04	Tertile 1	0.66 (0.40-1.07), P=9.6E-02
			Tertile 2	1.26 (0.84-1.88), P=5.3E-01
			Tertile 3	2.55 (1.83-3.56), P=3.1E-08
M3				
	172/1,884	1.93 (1.26-2.95), P=2.3E-03	Tertile 1	1.10 (0.57-2.12), P=7.7E-01
			Tertile 2	1.45 (0.82-2.55), P=2.0E-01
			Tertile 3	2.77 (1.73-4.46), P=2.7E-05
M3 (recessive)				
	21/106	1.19 (0.69-2.07), P=5.2E-01	Tertile 1	2.29 (0.64-8.12), P=2.0E-01
			Tertile 2	2.71 (0.97-7.59), P=5.6E-02
			Tertile 3	6.73 (2.59-17.5), P=8.8E-05

Supplementary Table 14: Genome-wide polygenic score (GPS) effect estimates for each *COL4A* gene under the M1 model. The odds ratios (ORs) were first derived using logistic regression models adjusted for age, sex, diabetes, batch, and genetic ancestry. Only UKBB data was included due to low case counts in the AoU dataset. The P-values (logistic regression Wald test) were two-sided and not adjusted for multiple testing.

Gene	Cases/controls	OR per SD (95% CI), P-value	GPS Tertile	OR (95% CI), P-value
COL4A3				
	12/211	1.37 (0.60-3.14), P=4.5E-01	Tertile 1	1.70 (0.65-4.43), P=2.8E-01
			Tertile 2	1.20 (0.36-3.98), P=7.7E-01
			Tertile 3	2.00 (0.70-5.72), P=1.9E-01
COL4A4				
	16/313	1.49 (0.73-3.03), P=2.7E-01	Tertile 1	0.86 (0.264-2.78), P=7.9E-01
			Tertile 2	1.69 (0.70-4.05), P=2.3E-01
			Tertile 3	2.23 (0.98-5.06), P=5.6E-02
COL4A3 or 4				
	28/524	1.91 (0.90-4.09), P=9.3E-02	Tertile 1	0.94 (0.41-2.18), P=8.8E-01
			Tertile 2	1.54 (0.78-3.02), P=2.1E-01
			Tertile 3	2.43 (1.31-4.52), P=5.0E-03
COL4A5				
	3/32	1.97 (0.18-21.10), P=5.7E-01	Tertile 1	3.46 (0.40-29.8), P=2.5E-01
			Tertile 2	1.35E-04 (2.6E-82-7.0E73), P=9.2E-01
			Tertile 3	11.0 (2.02-60.2), P=5.6E-03
