# Supplementary material for: Polygenic risk alters the penetrance of monogenic kidney disease
Source: Nat Commun. 2023 Dec 14;14:8318. doi: 10.1038/s41467-023-43878-9 (PMC10721887; doi:10.1038/s41467-023-43878-9)
Supplement: Supplementary file 3 — Description of Additional Supplementary Files [file 41467_2023_43878_MOESM3_ESM.pdf]

Description of Additional Supplementary Files

File name: Supplementary Data 1

Description: The list of qualifying variants for all three models utilized for ADPKD.

File name: Supplementary Data 2

Description: The list of qualifying variants for all three models utilized for COL4A-AN.

File name: Supplementary Data 3

Description: The summary statistics table of Meta-PheWAS of UKBB and AoU for ADPKD.

File name: Supplementary Data 4

Description: The summary statistics table of Meta-PheWAS of UKBB and AoU for COL4-AN.
