# Supplementary material for: Polygenic risk alters the penetrance of monogenic kidney disease
Source: Nat Commun. 2023 Dec 14;14:8318. doi: 10.1038/s41467-023-43878-9 (PMC10721887; doi:10.1038/s41467-023-43878-9)
Supplement: Supplementary file 8 — Reporting Summary [file 41467_2023_43878_MOESM8_ESM.pdf]

nature research

Corresponding author(s): Krzysztof Kiryluk

Last updated by author(s): Oct 27, 2023

Reporting Summary

Nature Research wishes to improve the reproducibility of the work that we publish. This form provides structure for consistency and transparency in reporting. For further information on Nature Research policies, see our Editorial Policies and the Editorial Policy Checklist.

Statistics

For all statistical analyses, confirm that the following items are present in the figure legend, table legend, main text, or Methods section.

n/a	Confirmed	
☐	☒	The exact sample size (*n*) for each experimental group/condition, given as a discrete number and unit of measurement
☐	☒	A statement on whether measurements were taken from distinct samples or whether the same sample was measured repeatedly
☐	☒	The statistical test(s) used AND whether they are one- or two-sided<br>*Only common tests should be described solely by name; describe more complex techniques in the Methods section.*
☐	☒	A description of all covariates tested
☐	☒	A description of any assumptions or corrections, such as tests of normality and adjustment for multiple comparisons
☐	☒	A full description of the statistical parameters including central tendency (e.g. means) or other basic estimates (e.g. regression coefficient) AND variation (e.g. standard deviation) or associated estimates of uncertainty (e.g. confidence intervals)
☐	☒	For null hypothesis testing, the test statistic (e.g. *F*, *t*, *r*) with confidence intervals, effect sizes, degrees of freedom and *P* value noted<br>*Give P values as exact values whenever suitable.*
☒	☐	For Bayesian analysis, information on the choice of priors and Markov chain Monte Carlo settings
☒	☐	For hierarchical and complex designs, identification of the appropriate level for tests and full reporting of outcomes
☐	☒	Estimates of effect sizes (e.g. Cohen's *d*, Pearson's *r*), indicating how they were calculated

Our web collection on statistics for biologists contains articles on many of the points above.

Software and code

Policy information about availability of computer code

Data collection	Electronic CKD Phenotype software is publicly available from the Phenotype Knowledge Database (PheKB, https://phekb.org/phenotype/chronic-kidney-disease).
Data analysis	The data was analyzed using open source statistical software packages including: R version 4.2.0 (CRAN), PLINK v. 1.9, Hail, EAGLE v.2, Minmac4, vcftools, and FlashPCA.

For manuscripts utilizing custom algorithms or software that are central to the research but not yet described in published literature, software must be made available to editors and reviewers. We strongly encourage code deposition in a community repository (e.g. GitHub). See the Nature Research guidelines for submitting code & software for further information.

Data

Policy information about availability of data

All manuscripts must include a data availability statement. This statement should provide the following information, where applicable:

- Accession codes, unique identifiers, or web links for publicly available datasets
- A list of figures that have associated raw data
- A description of any restrictions on data availability

The UKBB genotype and phenotype data are available through the UKBB web portal at https://www.ukbiobank.ac.uk/. The AoU genotype, WGS, and phenotype data are available through the AoU researcher workbench at https://www.researchallofus.org/data-tools/workbench/.

Field-specific reporting

Please select the one below that is the best fit for your research. If you are not sure, read the appropriate sections before making your selection.

☒ Life sciences ☐ Behavioural & social sciences ☐ Ecological, evolutionary & environmental sciences

For a reference copy of the document with all sections, see nature.com/documents/nr-reporting-summary-flat.pdf

Life sciences study design

All studies must disclose on these points even when the disclosure is negative.

Sample size	We utilized all case-control data available through UKBB and AoU without excluding any cohorts or studies.
Data exclusions	We included all available individuals in our analysis that had 1) adequate phenotype information to define case/control status, 2) whole exome or whole genome sequence data and 3) SNP genotype data available for analysis. No additional exclusion criteria were used.
Replication	We have tested the effects of GPS in two large cohorts with both sequence and SNP array data available, UKBB and All of US. We then meta-analyzed the results. We could not identify additional replication datasets of sufficient sample size to enable independent replication.
Randomization	Randomization was not required, since our study design involved analysis of observational data rather than an intervention.
Blinding	Blinding was not required, since our study design involved analysis of observational data.

Reporting for specific materials, systems and methods

We require information from authors about some types of materials, experimental systems and methods used in many studies. Here, indicate whether each material, system or method listed is relevant to your study. If you are not sure if a list item applies to your research, read the appropriate section before selecting a response.

Materials & experimental systems

n/a	Involved in the study
☒	☐ Antibodies
☒	☐ Eukaryotic cell lines
☒	☐ Palaeontology and archaeology
☒	☐ Animals and other organisms
☐	☒ Human research participants
☒	☐ Clinical data
☒	☐ Dual use research of concern

Methods

n/a	Involved in the study
☒	☐ ChIP-seq
☒	☐ Flow cytometry
☒	☐ MRI-based neuroimaging

Human research participants

Policy information about studies involving human research participants

Population characteristics	Two large cohort studies (UKBB and AoU) were included in the analysis, and detailed characteristics of study participants are provided in the manuscript with relevant references. Demographic characteristics pertinent to this study are also summarized for each cohort in Table 1, including ancestry, age, sex, diagnosis of diabetes, and CKD case status.
Recruitment	No active recruitment took place specifically for this study. The analysis involved only existing genetic cohorts.
Ethics oversight	This work was approved by the Columbia IRB under protocol number IRB-AAAC7385. All participants provided informed consent to participate in genetic studies.

Note that full information on the approval of the study protocol must also be provided in the manuscript.
